# Diminishing of *Helicobacter pylori* adhesion to *Cavia porcellus* gastric epithelial cells by BCG vaccine mycobacteria

**DOI:** 10.1038/s41598-023-43571-3

**Published:** 2023-09-28

**Authors:** Weronika Gonciarz, Maciej Chyb, Magdalena Chmiela

**Affiliations:** 1 Department of Immunology and Infectious Biology, Institute of Microbiology, Biotechnology and Immunology, Faculty of Biology and Environmental Protection, University of Lodz, Banacha 12-16, 90-237 Lodz, Poland; 2 Department of Molecular Microbiology, Faculty of Biology and Environmental Protection, University of Lodz, Lodz, Poland; 3 Bio-Med-Chem Doctoral School of the University of Lodz and Lodz Institutes of the Polish Academy of Sciences, Lodz, Poland

**Keywords:** Cell adhesion, Cellular imaging, Mechanisms of disease

## Abstract

*Mycobacterium bovis* onco-BCG bacilli used in immunotherapy of bladder cancer are candidates for training of immune cells towards microbial pathogens. Increasing antibiotic resistance of gastric pathogen *Helicobacter pylori* (Hp) prompts the search for new anti-Hp and immunomodulatory formulations. Colonization of gastric mucosa by Hp through mucin 5 AC (MUC5AC) ligands could potentially be a therapeutic target. The aim of this study was to examine the ability of onco-BCG mycobacteria to reduce Hp adhesion to gastric epithelial cells using *Cavia porcellus* model. Animals were inoculated *per os* with 0.85% NaCl, Hp alone, onco-BCG alone or with onco-BCG and Hp. After 7/28 days Mucin5AC and Hp binding to gastric epithelium were assessed in gastric tissue specimens by staining with anti-Mucin5AC and anti-Hp antibodies, respectively, both fluorescently labeled. Primary gastric epithelial cells were treated ex vivo with live Hp or Hp surface antigens (glycine extract or lipopolysaccharide) alone or with onco-BCG. In such cells MUC5AC and Hp binding were determined as above. Mycobacteria reduced the amount of MUC5AC animals infected with Hp and in gastric epithelial cells pulsed in vitro with Hp components. Decrease of MUC5AC driven in cell cultures in vitro and in gastric tissue exposed ex vivo to mycobacteria was related to diminished adhesion of *H. pylori* bacilli. Vaccine mycobacteria by diminishing the amount of MUC5AC in gastric epithelial cells may reduce Hp adhesion.

## Introduction

Local infection caused in humans by Gram-negative *Helicobacter pylori* (*H. pylori*) bacteria in the stomach or duodenum if is not eradicated can induce excessive inflammatory response, which may result with chronic gastritis, gastric or duodenal ulcers, mucosa associated lymphoid tissue (MALT) lymphoma or gastric cancer^1–3^.On average about 50% of population is infected with these bacteria, although the infection rate can be as high as 80–90%. About 20% *H. pylori* carriers develop symptoms. During chronic *H. pylori* infection, various virulence factors of these bacteria may diminish integrity of gastric barrier due to elevation of oxidative stress and cell apoptosis^4,5^. Colonization of gastric mucosa by *H. pylori* depends on motility, production of urease, which neutralizes gastric pH, and various adhesins facilitating the binding of *H. pylori* to gastric epithelial cells. Several *H. pylori* adhesins have been identified, including: the blood group antigen-binding adhesin A (BabA), sialic acid binding adhesin (SabA), interacting with Lewis b and sialylated Lewis X, respectively, adherence-associated lipoprotein A (AlpA) and B (AlpB), *Helicobacter* outer membrane protein Z (HopZ) and A (OpiA), proteins binding the host extracellular matrix (ECM) or Lewis X/Y determinants present in *H. pylori* lipopolysaccharide (LPS)^6–11^. In our previous study we showed that *H. pylori* and their soluble compounds increased the production of mucin 5AC (MUC5AC) and deposition of LeX/Y in the primary gastric epithelial cells of *Cavia porcellus*, and these effects were related to enhanced cell adhesion of these bacteria. The above results may suggest that *H. pylori* driven modulation of mucin production may facilitate colonization of gastric mucosa by these bacteria^12^. The infection becomes chronic due to the ability of *H. pylori* to modulate the effector mechanisms of immunocompetent cells, including phagocytes^13–15^, natural killer (NK) cells^16^, and T lymphocytes^17^. These *H. pylori* properties may establish persistent infection and influence the clinical outcome of infection^18,19^. According to Word Health Organization (WHO) reports *H. pylori* resistance to antibiotics used for treatment of infection has reached alarming levels worldwide, which has a great influence on treatment effectiveness^20^. It has been recommended by WHO to search for new formulations for supporting therapy towards *H. pylori*. Different immunomodulatory compounds, which are able to enhance the immune mechanisms of the host and limit the colonization of *H. pylori* have been considered. In this study we were focusing on Bacillus Calmette-Guérin (BCG), a live attenuated anti-tuberculosis vaccine mycobacteria, to see whether they can interfere with *H. pylori* adhesion to gastric epithelial cells. BCG vaccine containing attenuated *Mycobacterium bovis* is intensively evaluated as bacterial immunomodulator in terms of inducing non-specific cross-protection against pathogens unrelated to the target disease, such as *Candida albicans* and *Staphylococcus aureus*^21^ on the way of the immune training^22^**.** *Mycobacterium bovis* onco-BCG formulation has been used successfully as an immunomodulator supporting the therapy of bladder cancer^23^. Several studies revealed the possibility of diminishing *H. pylori* gastric colonization by probiotic bacteria^24^. However, it is unknown whether *M. bovis* onco-BCG administered *per os* can interfere with *H. pylori* adhesion to gastric mucosa. Our preliminary results show that onco-BCG increase the phagocytic activity of human THP-1 derived macrophages, which was affected by *H. pylori* (unpublished data). In this study we used in vivo model of *Cavia porcellus* (guinea pig), characterized by us in terms of immune response during an experimental *H. pylori* infection^25^. Animals were inoculated *per os* with *H. pylori* alone, *M. bovis* onco-BCG alone or simultaneously with *H. pylori* and onco-BCG mycobacteria. In animals treated as above the MUC5AC was determined in conjunction with assessment of *H. pylori* adhesion by staining of gastric specimens with fluorescently labeled ani-MUC5AC or anti-*H. pylori* antibodies, respectively. We also used a model of *Cavia porcellus* primary gastric epithelial cells, which were exposed to *H. pylori* components of these bacteria and/or onco-BCG bacilli in vitro to confirm the influence of mycobacteria on the process of *H. pylori* adhesion to gastric epithelial cells in conjunction with MUC5AC level.

## Results

**Figure 1:**
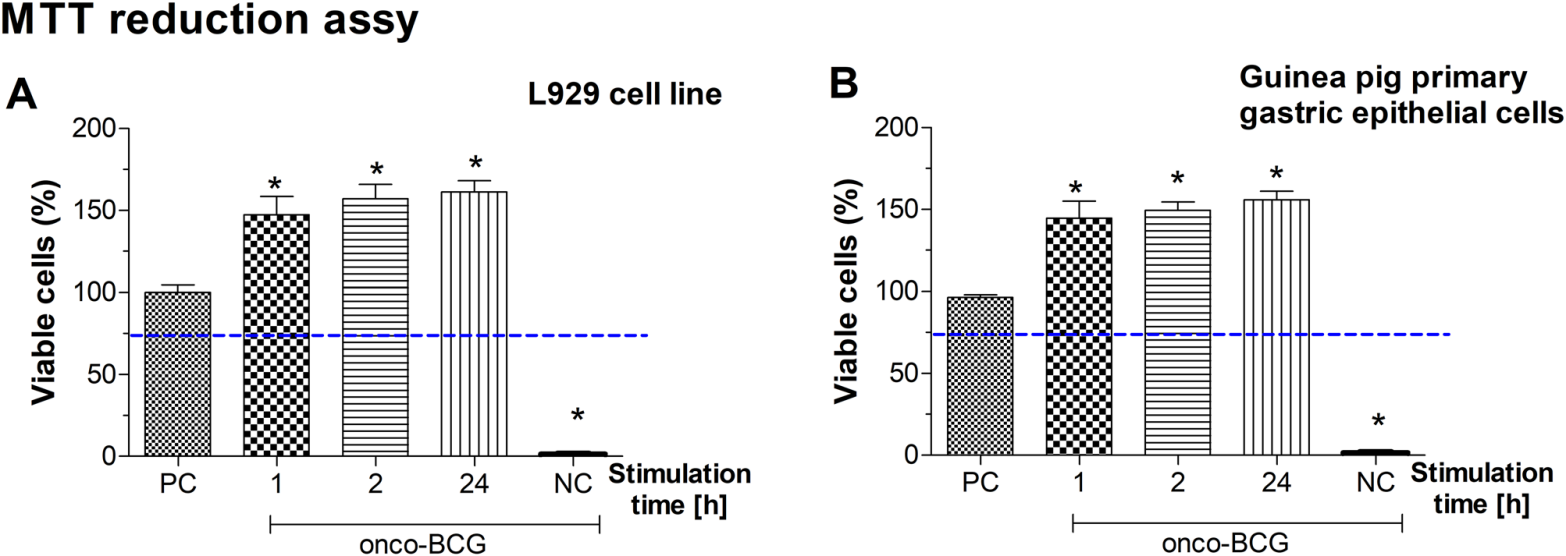
Biocompatibility of *M. bovis* onco-BCG. The viability of mouse fibroblasts L929 (**A**) or guinea pig primary gastric epithelial cells (**B**) treated with onco-BCG mycobacteria (onco-BCG). The percent of cells, which were able to reduce tetrazolium salt (3-(4,5-Dimethylthiazol-2-yl)-2,5-Diphenyltetrazolium Bromide) (MTT) was determined. NC-negative control (cells treated with 0.03% H_2_O_2_-non viable cells), PC-positive control (cells in medium alone, 100% cell viability). Results are showed as mean ± standard error of mean (SEM). Three experiments were performed in triplicates for each experimental variant. The blue line indicates the minimal percentage of viable cells (70%) required to confirm the onco-BCG as non-cytotoxic in vitro. Statistical analysis was performed using the nonparametric U Mann–Whitney test with significance, p < 0.05 (*unstimulated cells vs. cells exposed to onco-BCG).

We were focusing on answering the question whether onco-BCG vaccine bacilli influence the adhesion of *H. pylori* to gastric epithelial cells on the guinea pig model taking into account MUC5AC as target host molecule for mycobacteria. According to the ISO norm (ISO 10993-5:2009) we checked the cytocompatibility of onco-BCG mycobacteria against L929 mouse fibroblast and guinea pig primary gastric epithelial cells in vitro, in MTT reduction assy. Onco-BCG mycobacteria did not induce the cytotoxic effects towards tested cells after stimulation for 1, 2 or 24 h. The cell viability was higher than 70%, meeting the biological safety standard (Fig. 1). The amount of viable cells in cell cultures exposed to mycobacteria was higher than in control cell cultures propagated in culture medium alone indicating that onco-BCG bacilli were able to stimulate a cell growth.

**Figure 2:**
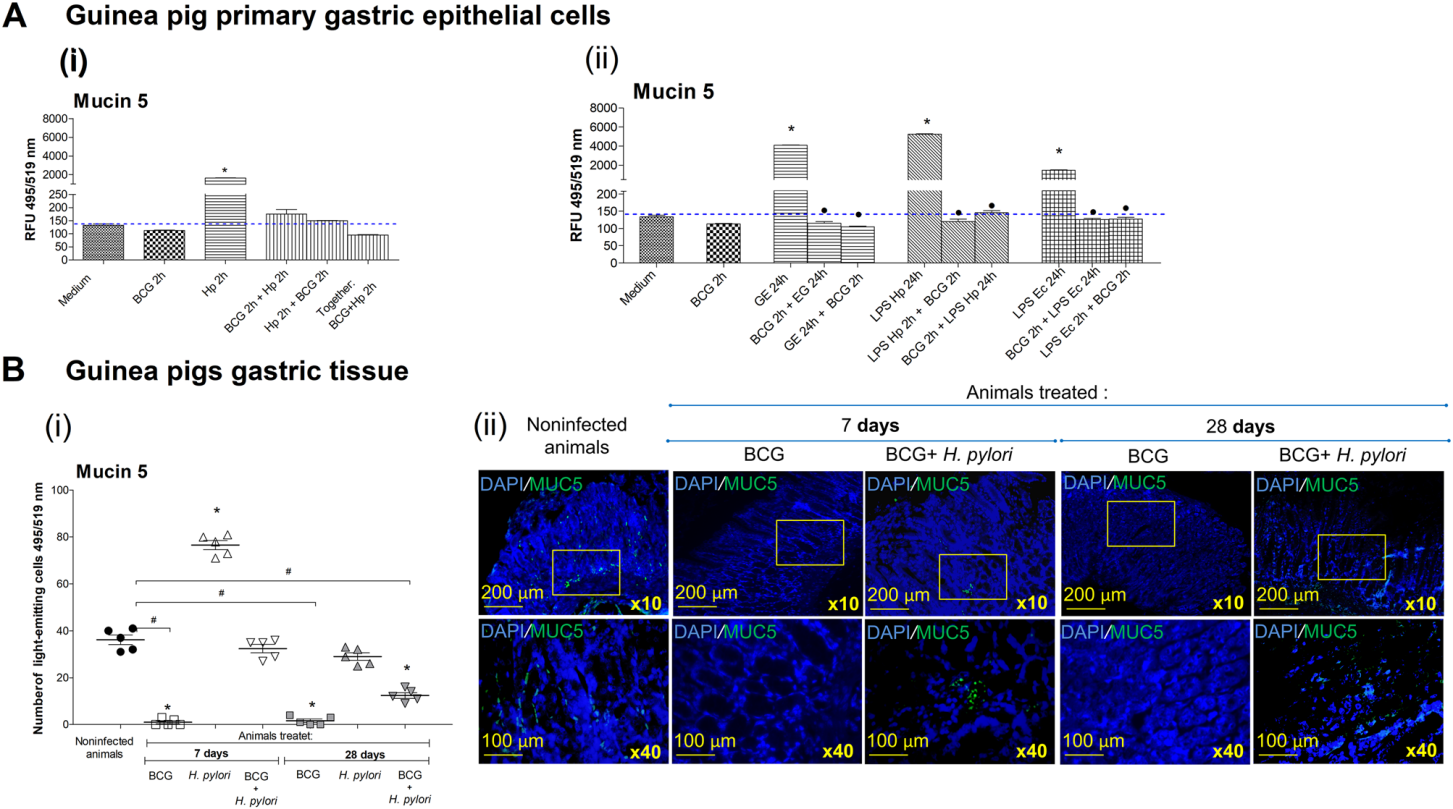
Detection of Mucin 5AC (Mucin 5AC) in vitro (**A**) in cell cultures of guinea pig primary gastric epithelial cells or in vivo (**B**) in guinea pig gastric tissue. Evaluation of Mucin 5AC in vitro (**A**) in cells exposed to onco-BCG and/or *H. pylori* (i), to *H. pylori* components and/or onco-BCG mycobacteria (BCG) (ii) or in vivo (**B**) in gastric tissue of control animals or animals inoculated with *H. pylori* or onco-BCG alone or first with onco-BCG and then with *H. pylori*. Mucin 5AC was assessed by measurement of the fluorescence intensity following staining of cells with mouse anti-MUC5AC antibodies and with secondary anti-murine immunoglobulin antibodies labeled with fluorescein isothiocyanate (FITC). Cell nuclei were stained with 4′,6-diamidino-2-fenilindol-DAPI. (**A**) The fluorescence intensity was measured in multifunctional reader SpectraMaxi3 at the appropriate wavelength: 495 nm (excitation), 519 nm (emission). Mean ± standard error of mean (SEM) are shown. Statistical significance at p < 0.05 in the non-parametric U-Mann–Whitney or Kruskal–Wallis test. *Cells treated with *H. pylori* or components of these bacteria *vs* untreated cells; ● cells treated with *H. pylori* components or *H. pylori* and onco-BCG as compared to cells treated with *H. pylori* components alone or with these bacteria. Hp-*H. pylori*; Ec-*Escherichia coli,* RFU-relative fluorescence unit, GE-glycine extract, LPS-lipopolysaccharide. (**B**) (i) The number of luminous cells was obtained with ImageJ 1.48v software at 495 nm (excitation), 519 nm (emission). Mean ± standard error of mean (SEM) are shown. (ii) Representative images of gastric tissue of guinea pigs not receiving onco-BCG bacilli and not infected with *H. pylori,* receiving onco-BCG bacilli, receiving onco-BCG bacilli and then *H. pylori,* 7 or 28 days after inoculation with *H. pylori* (n = 5). Sections were immunohistochemically stained for Mucin 5AC with specific fluorescently labeled antibodies and imaged under a confocal microscope (Leica TCS SPE) at wavelengths for FITC: 495 nm (excitation), 519 nm (emission) or for DAPI: 345 nm (excitation), 455 nm (emission) (10× and 40× magnification). Statistical significance for p < 0.05 in the non-parametric Mann–Whitney or Kruskal–Wallis U test. *Animals infected with *H. pylori vs* uninfected animals, # animals receiving onco-BCG and infected with *H. pylori vs* animals receiving onco-BCG or infected with *H. pylori*.

In cell cultures of guinea pig primary gastric epithelial cells treated with BCG bacilli alone (MOI 10:1) we could not see the increased amount of cellular MUC5AC as showed by immunostaining with anti-MUC5AC antibodies, however, MUC5AC was significantly increased in cells exposed to live *H. pylori,* p < 0.05 in Mann–Whitney *U* test (Fig. 2Ai). MUC5AC was also increased in cells in cell cultures containing bacterial components: *H. pylori* antigenic complex called a glycine acid extract-GE and *H. pylori* LPS, or LPS *Escherichia coli* (Fig. 2Aii). By comparison MUC5AC was reduced in cells treated simultaneously in cell cultures in vitro with onco-BCG bacilli and *H. pylori*, in cell cultures primed with *H. pylori* and then treated with onco-BCG or in cell cultures primed with onco-BCG and then exposed to *H. pylori* (Fig. 2Ai). The amount of MUC5AC, which was increased in the milieu of *H. pylori* soluble components, GE or LPS, was reduced in cells primed with onco-BCG and then exposed to *H. pylori* components or in cells primed with these components and then treated with onco-BCG (Fig. 2Aii). The neutralizing effect of onco-BCG towards MUC5AC was dependent on the presence of mycobacteria in the cell culture but not on the order in which the cells were exposed to mycobacteria.

For in vivo study we used the guinea pig model, which was characterized by us in terms of *H. pylori* colonization by histological examination (detection of *Helicobacter* like organisms—HLO, evaluation of inflammatory response), molecular testing—detection of gene encoding UreC subunit of urease and gene encoding CagA protein, and serological assessment of anti-*H. pylori* antibodies in serum samples by the Enzyme-linked Immunosorbent Assay (ELISA)^25^. In gastric tissue specimens from *H. pylori* infected animals, but not in control group, which were routinely stained by Giemsa stain solution or Mayer hematoxylin and eosin, the *Helicobacter*-like organisms-HLO (+++) and infiltration of gastric tissue mainly by granulocytes was showed after 7 or 28 days from inoculation of animals with *H. pylori* (5/5 and 5/5, respectively)*.* In gastric tissue of *H. pylori* infected animals the infiltration of lymphocytes was also noticed, which however was more pronounced after 28 (++) than after 7 days (+) from inoculation with *H. pylori*. All tissue sections were also positive for *ureC* and *cagA*. In animals receiving BCG and next infected with *H. pylori* the inflammation markers after 7 or 28 days from inoculation with *H. pylori* were similar to those in the animals receiving only *H. pylori*. We could not see the important difference in the level of HLO between *H. pylori* group *vs* onco-BCG and *H. pylori* group as well as in detection of *ureA* and *cagA*. There was also no significant difference in the level of anti-*H. pylori* antibodies between these two groups. The gastric tissue specimens were positive for urease and the isolates from the gastric tissue from *H. pylori* exposed animals demonstrated the urease, catalase and oxidase activity^25^.

In vivo, the amount of MUC5AC was higher in the gastric tissue cells of *H. pylori* infected animals 7 days after oral administration of these bacteria, than in uninfected animals (Fig. 2Bi, ii). The amount of MUC5AC in the gastric tissue cells of guinea pigs 28 days from inoculation with *H. pylori* was not significantly increased. In animals receiving onco-BCG bacilli, the amount of MUC5AC was significantly lower than in control animals,* p* < 0.05 in Kruskal–Wallis test. Administration of onco-BCG mycobacteria to animals prior inoculation of them with *H. pylori* resulted in reduction of MUC5AC in the gastric tissue cells compared to animals receiving only *H. pylori*. The difference was statistically significant in the chronic phase of infection (28 days after *H. pylori* administration),  *p* < 0.05 in Kruskal–Wallis test (Fig. 2Bi, ii).

**Figure 3:**
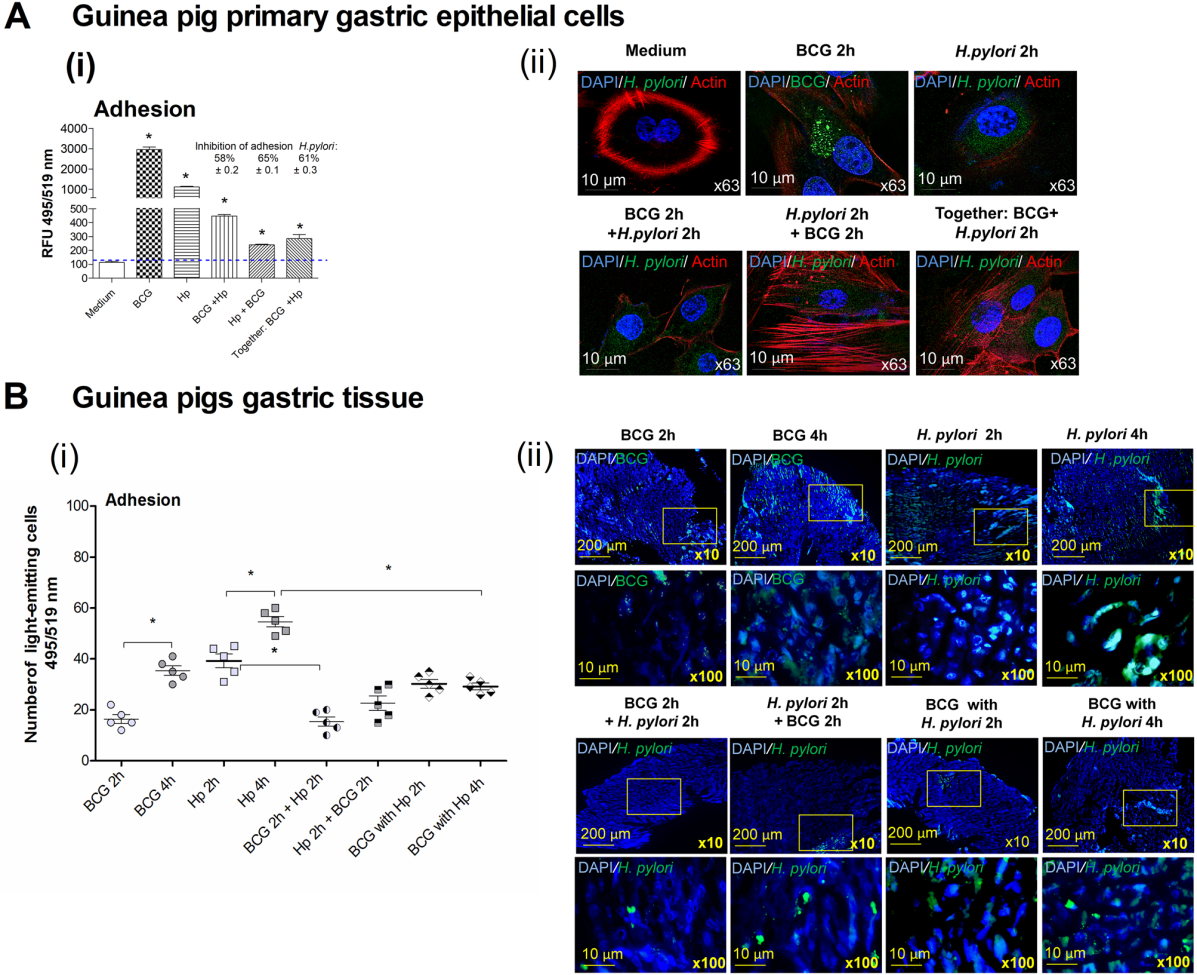
Modulation of *H. pylori* adhesion to primary gastric epithelial cells of guinea pig (**A**) or to gastric tissue specimens of guinea pig (**B**) by onco-BCG mycobacteria. Binding of *H. pylori* to guinea pig primary gastric epithelial cells (**A**) was assessed by staining with anti-*H. pylori* antibodies labeled with fluorescein isothiocyanate (FITC) while onco-BCG mycobacteria (BCG) using BactLight staining procedure. Cell nuclei were stained with 4′,6-diamidino-2-fenilindol-DAPI while the cytoskeleton with Texas-Red-phalloidin. (i) *H. pylori* adhesion was expressed as fluorescence intensity measured in a multifunctional reader SpectraMax i3 at wavelengths for FITC 495 (excitation) and 519 (emission). The results are presented as mean ± standard error of mean (SEM). Statistical significance for *p* < 0.05 in the non-parametric Mann–Whitney or Kruskal–Wallis U test. *Cells treated with onco-BCG or *H. pylori vs* control cells in medium only, ● cells treated with onco-BCG and *H. pylori vs* cells treated with *H. pylori* only. (ii) representative images showing the adherence of *H. pylori* rods to primary gastric epithelial cells of the guinea pig under a confocal microscope at appropriate wavelengths: for FITC 495 nm (excitation) and 519 (emission), for DAPI 345 (excitation) and 455 (emission), and for phalloidin 591 nm (excitation) and 608 nm (emission), magnification 64×. (**B**) Binding of *H. pylori* to guinea pig gastric tissue was assessed by staining of specimens with anti-*H. pylori* antibodies labeled with fluorescein isothiocyanate (FITC). Onco-BCG mycobacteria were stained with BactLight. Cell nuclei were stained with DAPI. Cells were imaged under the confocal microscope (Leica TCS SPE). (i) the number of glowing cells was calculated using ImageJ version 1.48 software at 495nm (excitation) and 519nm (emission) FITC wavelengths. The results are presented as mean ± standard error of mean (SEM). (ii) representative images of gastric tissue with adjacent *H. pylori* bacilli from a fluorescence microscope, at 495 nm (excitation) and 519 nm (emission) for FITC, 345 nm (excitation) and 455 nm (emission), (10, 100 × magnification) for DAPI Statistical significance for* p * < 0.05 in the non-parametric U Mann–Whitney or Kruskal–Wallis test. Tissue treated: *H. pylori and onco-BCG-versus *H. pylori*, ^#^*H. pylori* or onco-BCG for 2 h and 4 h.

To determine whether the amount of MUC5AC, which was downregulated in gastric tissue cells of guinea pigs by onco-BCG bacilli was related to the efficiency of *H. pylori* binding we developed in vitro study. For this purpose, guinea pig primary gastric epithelial cells or gastric specimens isolated from *H. pylori* uninfected guinea pig were incubated ex vivo with live *H. pylori* alone, with onco-BCG bacilli alone or with *H. pylori* and onco-BCG bacilli. Both *H. pylori* and *M. bovis* onco-BCG attached to gastric epithelial cells, which were exposed to these bacteria in cell culture for 2h. The fluorescence intensity of cells treated with onco-BCG bacilli or *H. pylori* alone was significantly higher than the fluorescence intensity of cells grown in medium alone (control),  *p* < 0.05 in Mann–Whitney U test (Fig. 3Ai, ii). Adhesion of *H. pylori* to primary gastric epithelial cells was significantly ( *p* < 0.05 in Mann–Whitney U test) inhibited by onco-BCG mycobacteria, which were administered into the cell culture before the exposure of cells to *H. pylori* or when *H. pylori* and onco-BCG were added simultaneously (Fig. 3Ai, ii).

Both *H. pylori* and onco-BCG mycobacteria adhered to the gastric tissue specimen of guinea pig, the binding efficiency was better after exposure of cells to these bacteria for 4 h than for 2 h (Fig. 3Bi, ii). In addition, onco-BCG mycobacteria significantly (*p* < 0.05, in Kruskal–Wallis test) diminished the binding of *H. pylori* to guinea pig gastric tissue in all variants of tissue exposure to *H. pylori* and onco-BCG bacilli (Fig. 3Bi, ii). However, the best inhibitory effect of onco-BCG mycobacteria towards the adherence of *H. pylori* to gastric tissue was demonstrated for tissue sections, which were primed for 2 h with onco-BCG bacilli and then incubated with *H. pylori.*

## Discussion

The improving of knowledge about the nature of *H. pylori* interactions with the gastric epithelium and immune cells may help to find new components potentially therapeutic, which would effectively prevent the colonization of the gastric mucosa by these bacteria and to support the activity of immunocompetent cells towards *H. pylori.* For these purposes different in vitro cell models and in vivo animal models, including the model of *Cavia porcellus* (guinea pig) have been developed^25,26^. *Cavia porcellus* is the promising animal model for studying the course of various bacterial infections. The anatomy and physiology of the stomach is similar in guinea pig and in human. Furthermore, guinea pig possess similar mechanisms of immune responses, including the production of pro-inflammatory IL-8 and the development of specific adaptive immunity, cellular and humoral^27^. Moreover, guinea pigs are not naturally colonized with *H. pylori*^26^.

The binding of *H. pylori* to gastric mucosa is driven by different bacterial adhesins, including the BabA or SabA, interacting with human antigens, Le b or sialylated Le X blood group antigens, respectively^6,8,27–30^. The open question is whether it would be possible to prevent or diminish *H. pylori* colonization with *per os* administration of different safe bacteria on the similar way as probiotic bacteria^24^. Recently, *M. bovis* BCG vaccine mycobacteria have gained a lot of interest due to their immunomodulating properties, which upregulate the activity of innate immune cells, monocytes, macrophages and natural killer cells against infectious agents. BCG mycobacteria induce non-specific cross-protection against pathogens unrelated to the target disease, such as *C. albicans* and *S. aureus*^21^. It has been suggested that mortality and deleterious effects due to SARS-Cov-2 viral infection were lower in BCG vaccinated than non-vaccinated populations^31^. Furthermore, *M. bovi*s onco-BCG formulation has been used successfully in the immunotherapy of bladder cancer and melanoma^23^.

The stomach has a two layered mucus system, composed of an inner, attached mucus and an outer, unattached, loose mucus layer, both built by MUC5AC mucin produced by superficial epithelium. The inner mucus layer built around MUC5AC, acts as a diffusion barrier for hydrochloric acid. The surface epithelial cells secrete bicarbonate, creating pH gradient from the acidic lumen to neutral pH at the cell surface. Surface mucus hydrogel protects the underlying gastric epithelium by trapping pathogens^32^, however, gastric pathogen *H. pylori* during the first stage of infection occupy the gastric mucus layer using host components, which play a role of early receptors for *H. pylori* adhesins before the later interaction of these bacteria with epithelium^30,33,34^

Previously, using the model of guinea pig experimentally infected with *H. pylori,* we showed that increased MUC5AC production and elevated deposition of Lewis X antigens in the gastric tissue was correlated with significantly increased colonization of *H. pylori*^12^. In primary gastric epithelial cells sub-cultured in vitro for 24 h with *H. pylori* soluble components: GE, CagA protein, sub-unite A of urease (UreA) or LPS, or after 2 h exposure of cells to live *H. pylori* the production of MUC5AC was enhanced. This was correlated with elevated adhesion of *H. pylori* to gastric epithelial cells. Blocking of MUC5AC with specific anti-MUC5AC antibodies resulted with diminished adhesion of *H. pylori*^12^.

In this study we asked whether onco-BCG vaccine mycobacteria can safely modulate the binding of *H. pylori* to the gastric tissue and whether MUC5AC, which is involved in *H. pylori* binding may be a target for BCG bacilli. During gastric passage, mycobacteria administered *per os* may potentially compete with *H. pylori* for binding sites in gastric mucus or on the surface of gastric epithelial cells since they can resist in acidic milieu of the stomach. Similarly to *H. pylori,* mycobacteria produce urease, which hydrolyzes urea into carbon dioxide and ammonia, neutralizing the acidic pH of gastric juice. Urease also protects mycobacteria against intracellular degradation^35^. Mycobacterial species produce numerous surface adhesins, including heparin-binding hemagglutinin (hbhA), laminin or fibronectin binding compounds, which potentially may facilitate colonization of gastric epithelial cells by these bacteria^36,37^. It has been shown that *M. avium* invade the intestinal mucosa primarily by interacting with enterocytes^38^. The surface localization of DNA-binding protein 1 (MDP1) in cells of mycobacteria and its capacity to bind hyaluronic acids, heparin or chondroitin sulphate imply a possible function as adhesion molecules facilitating binding and entry of mycobacteria into epithelial cells^39^. Also intelectin-1 deposited on various cell surfaces, including different epithelial cell lines and macrophages is involved in capture of *M. bovis*^40^. Mycobacteria produce proteinase, thioredoxin, phospholipase C^41^ and mucinases^42^, which potentially may degrade MUC5AC. Periodic acid-Schiff (PAS) and Alcian blue (AB) are standard mucin histochemical stains that react with monosaccharides. However, these stains are not intrinsically specific for mucins, and may also stain of non-mucin glycoproteins, including proteoglycans an d glycogen, a cytoplasmic constituent of cells that is often difficult to distinguish morphologically from mucins^43^, and due to this more specific staining by immunostaining with mucin-specific antisera is recommended. In this study we stained mainly a gastric cell MUC5AC in guinea pig primary gastric cells in cell cultures in vitro or in guinea pig gastric tissue using mouse monoclonal antibodies cross-reacting with human and rodent MUC5C.

In this study the precise quantification of gastric tissue colonization with *H. pylori*, in our guinea pig model utilizing guinea pigs bred and fed in standard conditions, by colony forming unit method (CFU), and the modulation of colonization with these pathogens by mycobacteria using CFU was problematic due to reach commensal microflora in the stomach of guinea pigs. In the present study, we used an immunohistochemical staining to see whether the exposure of guinea pig gastric tissue in vivo or gastric cells under culture conditions to onco-BCG mycobacteria may diminish the *H. pylori* driven MUC5AC production and whether mycobacteria may influence the adhesion of *H. pylori* to gastric epithelial cells in vitro or ex vivo using a guinea pig gastric tissue. Our preliminary results suggest the possibility of reducing of adherence of *H. pylori* to gastric cells or gastric tissue in conjunction with modulation of the amount of MUC5AC by *M. bovis* BCG.

As we showed in this study, *M. bovis* BCG bacilli diminished *H. pylori* adhesion to gastric epithelial cells in vitro or to gastric tissue exposed to onco-BCG ex vivo by MUC5AC modulation. Gastric epithelial cells treated with onco-BCG before/after or simultaneously with live *H. pylori* or their soluble components, GE or LPS, showed the reduction of MUC5AC staining. In animals receiving onco-BCG, the amount of stained MUC5AC was lower than in control animals. Oral administration of onco-BCG to animals prior to inoculation with *H. pylori* resulted in reduction of MUC5AC staining compared to animals receiving only *H. pylori*. Potentially onco-BCG mycobacteria can compete with *H. pylori* for MUC5AC binding sites or they can decrease the production of MUC5AC or eventually cause its degradation. It is also possible that complexes of BCG-onco-MUC5AC are shed from the surface of gastric epithelial cells. Thus in the gastric mucosa onco-BCG mycobacteria may upregulate and utilize the same molecules for binding as *H. pylori* do^44^. It has been recently shown that *M. tuberculosis* infection upregulates sialyl LewisX expression in the lung epithelium^45^ and that *M. bovis* infection led to increased expression of specific Lewis epitopes in N-glycans on macrophages^46^. Glycans are important mediators in the colonization of host tissues by microorganisms and are important molecules for cellular signaling in response to infection^47^. It is possible that *M. bovis* urease, adhesins combined with poor mobility of mycobacteria and the ability to biofilm formation^48^, may result in blocking of *H. pylori* binding sites in the gastric epithelial cells. However, secreted MUC5AC, during formalin fixation of gastric cells or the tissue to stain for MUC5AC might have been partially lost. It is possible that BCG treatment of primary gastric cells or gastric tissue may in fact increase mucus secretion with a corresponding depletion of MUC5AC staining in cells. Increased MUC5AC secretion in response to *M. bovis* BCG may lead to more *H. pylori* bacteria in the adherent mucus layer and less adhesion to epithelial cells.

However, the potential mechanism of reduction of gastric mucus production by mycobacteria should not be omitted. It has been shown that intranasal inoculation of BALB/c mice with BCG strain reduced lung pathology, and airway mucus production^49,50^. The study by Middleton et al., revealed that mycobacteria adhere specifically to mucus and extracellular matrix in organ cultures of human respiratory mucosa. The number of adhering mycobacteria was markedly reduced after incubation these bacteria with mucins, MUC5AC or MUC5B, however, mycobacterial infection did not increase mucin production by the organ culture^51^

Our preliminary results in the guinea pig model, show that onco-BCG bacilli may modulate the amount of MUC5AC in gastric tissue, which potentially may result in control of *H. pylori* adhesion. Considering our data obtained in this study and the above-mentioned results of other authors, further research is necessary to explain the intriguing result of modulation of gastric MUC5AC in conjunction with adhesion of *H. pylori* to gastric tissue. Our interesting data require further quantitative confirmation using in vivo model of guinea pig under conditions of *H. pylori* infection or exposure of animals to onco-BCG and then to *H. pylori*. For this purpose a quantitative molecular methods for this model are under development to show differences in the number of *H. pylori* bacilli in the gastric tissue of animals inoculated with *H. pylori vs* animals exposed to BCG and infected with *H. pylori*.

The obtained results also prompt for further study to determine the ability of onco-BCG formulation to modulate the activity of immunocompetent cells towards *H. pylori.* Potentially it can be achieved in the lower gastrointestinal tract where mycobacteria can persist temporary and be internalized by mucosal epithelial cells, including M cells, where the interaction with the immunocompetent cells is possible^42^. It has been revealed that internalization of mycobacteria by epithelial cells resulted in the production of tumor necrosis factor-α and granulocyte macrophage colony-stimulating factor, the cytokines which potentiate antimicrobial activity of macrophages^52,53^.

## Conclusion

In this study we showed that live *H. pylori* as well as soluble components of these bacteria were able to upregulate Mucin 5AC, which was related with successful colonization of guinea pig stomach by *H. pylori *in vivo or guinea pig derived primary gastric epithelial cells in vitro*.* Onco-BCG mycobacteria by modulation of MUC5AC in gastric cells were able to reduce *H. pylori* adhesion. These results suggest the possibility of controlling the development of *H. pylori* infection by onco-BCG mycobacteria on the colonization level. Further research is needed to deepen the mechanism of influence of onco-BCG vaccine mycobacteria on the course of *H. pylori* infection.

## Materials and Methods

### Ethical statement

All experiments involving animals were developed according to the Animal Research: Reporting of In Vivo Experiments (ARRIVE) guidelines and guidelines and regulations EU directive (Directive 2010/63/EU of the European Parliament and of the Council of 22 September 2010 on the protection of animals used for scientific purposes (Dz.U. L 276 z 20.10.2010, s. 33–79) and were approved by the Local Ethics Committee (LKE9) for Animal Experiments of the Medical University of Lodz, Poland, which was established by the Ministry of Science and Higher Education in Poland (Ethics Committee decision number: 58/ŁB45/2016. Both genders of three-month guinea pigs (five animals per group, respecting the 3R principle), free of pathogens, were bred and housed in the Animal House at the Faculty of Biology and Environmental Protection, University of Lodz (Poland), in accordance with Laboratory Animal Resources Commission standards The animals were kept in air-conditioned rooms at 20–24 °C in cages with free access to drinking water and food pellets ad libitum. They were exposed to a 12 h light/dark cycle.

### Bacterial stimulators

*Helicobacter pylori* reference strain CCUG 17874 (Culture Collection, University of Gothenburg, Gothenburg, Sweden), positive for vacuolating cytotoxin A (VacA) and cytotoxin associated gene A (CagA) protein was cultured under microaerophilic conditions according to the previously described procedure^5,12^. *H. pylori* reference strain as well as *Mycobacterium bovis* onco-BCG preparation (SYNTHAVERSE, Lublin, Poland), Brazilian Moreau substrain, were used in the cell study in vitro in the multiplicity of infection (MOI): 10:1. Bacterial surface components from the reference *H. pylori* strain were extracted using 0.2 M glycine buffer, pH 2.2, as previously described^54^. The glycine acid extract (GE) was evaluated for protein composition by sodium dodecyl sulphate polyacrylamide gel electrophoresis (SDS-PAGE), and a Western blotting (immunoblotting assay) (Milenia Blot *H. pylori*, DPC Biermann, GmbH, Bad Nanheim, Germany). For this purpose the reference serum samples from patients infected with *H. pylori* were used. Major proteins in GE recognized by the sera of patients are: 120 kDa, 80 kDa, between 66 and 42 kDa and 29–26 kDa. The protein content in GE was 600 μg/mL (NanoDrop 2000c Spectrophotometer, Thermo Fischer Scientific, Waltham, MA, USA). The GE sample contained < 0.001 EU mL of LPS as shown by the chromogenic *Limulus amebocyte* lysate test (Lonza, Walkersville, MD, USA). LPS from the reference *H. pylori* strain (obtained by courtesy of AP. Moran), was prepared by hot phenol-water extraction after pre-treatment of the bacterial biomass with protease. The crude LPS preparation was purified by RNAse, DNAse and proteinase K treatment and by ultracentrifugation, as previously described^55^. *E. coli* LPS derived from the O55:B5 strain (Sigma Aldrich, Saint Louis, MO, USA), was used as control. The concentration of *H. pylori* components was selected experimentally or used as previously described, and was equal to: GE 10 µg/mL, *H. pylori* LPS and *E.coli* LPS 25 ng/mL^4,5,12^.

### Guinea pig model

Three-month-old, male Himalayan guinea pigs (400–600 g), free of pathogens, were housed in the Animal House at the Faculty of Biology and Environmental Protection, University of Lodz (Poland), kept in cages with free access to drinking water and fed with standard chow. A total of 35 animals were used in the study (5 per study groups). The feeding of animals was discontinued 24 h before the beginning of the experimental procedure, with constant access of animals to drinking water. The animals were split into the following groups: control animals—uninfected (received 1 mL of 0.2 N NaHCO_3_ by oral route (without anesthesia), and then, after 5 min, 1 mL of *Brucella* broth with the addition of 10% fetal bovine serum (FBS) (without anesthesia). The procedure was repeated three times at two-day intervals. Animals infected with *H. pylori* received 1 mL of 0.2 N NaHCO_3_ orally (without anesthesia), and then after 5 min, 1 mL of the *H. pylori* suspension 1 × 10^10^ CFU/mL (without anesthesia). The inoculation procedure was repeated 3 times at two-day intervals. Animals inoculated with *M. bovis* onco-BCG (without anesthesia), and then infected with *H. pylori* received 1 mL of 0.2 N NaHCO_3_ by oral route(without anesthesia), and then, after 5 min, 1 mL of *M. bovis* onco-BCG with a density of 1 × 10^8^ CFU/mL (without anesthesia). After 3, 5, 7 days, the animals were infected with a suspension *of H. pylori* with a density of 1 × 10^10^ CFU/mL). Animals inoculated with *M. bovis* onco-BCG alone received 1 mL of 0.2 N NaHCO_3_ by oral route (without anesthesia), and then, after 5 min 1 mL of *M. bovis* onco-BCG with a density of 1 × 10^8^ CFU/mL (without anesthesia). After 3, 5, 7 animals received 1 mL of *Brucella* broth supplemented with 10% FCS (without anesthesia). The procedure was repeated three times at two-day intervals. After 7 or 28 days from receiving the last dose of bacteria (infected group), or animals in the control group, were euthanized according to a protocol approved by an ethics committee, and the gastric tissue was collected and prepared for analyses as previously described^5,12^. In brief, the gastric tissue from the *antrum* of the stomach was evaluated for the presence of *Helicobacter*-like organisms (HLO) after routine staining with Giemsa stain solution or Mayer hematoxylin and eosin. The amount of HLO was graded as: (+++), (++), (+). In the same tissue sections the inflammatory reaction was evaluated using the Sydney system criteria used for histological examination of gastric tissue samples. Fresh tissue specimens were examined for the activity of urease and isolates also for catalase and oxidase. Infection was also confirmed by detection of genes encoding UreC subunit of urease or CagA protein by qualitative polymerase chain reaction (PCR). In serum samples the level of anti-*H. pylori* antibodies was assessed by the laboratory enzyme linked immunosorbent assay (ELISA) using the complex of *H. pylori* antigens called a glycine acid extract (GE).

### Isolation and growing of guinea pig primary gastric epithelial cells

Guinea pig primary gastric epithelial cells were isolated from animals, the usage of which was approved by the ethics committee as previously described^5,12^. Briefly, cells were cultured under standard conditions (37 °C, 5% CO_2_) in a mixture of DMEM and Hams F-12 media (ratio 1:1; Sigma-Aldrich, Saint Louis, MI, USA), supplemented with 10% fetal calf serum (FCS), 1% (N-2-hydroxyethylpiperazineN-2-ethane sulfonic acid) (HEPES), penicillin (100 U/mL), streptomycin (100 µg/mL), amphotericin B (0.025 µg/mL), l-glutamine (2 mM/mL), epidermal growth factor (Sigma Aldrich, Saint Louis, MI, USA) 0.01 µg/mL, and 0.005% dexamethasone Sigma Aldrich, Saint Louis, MI, USA).

### L-929 cell culture

The reference L-929 (CCL-1™) mouse fibroblasts were obtained from the American Type Culture Collection (purchased in ATCC, Rockville, Manassas, VA, USA). Cells were grown in RPMI (Roswell Park Memorial Institute)-1640 medium supplemented with 10% heat-inactivated FCS, 100 U/mL penicillin, 100 U/mL streptomycin, 2 mM/mL l-glutamine at 37 °C (c RPMI), (all from Sigma St. Louis, MI, USA), in an atmosphere of cell culture incubator, containing 5% CO_2_. Cells were passaged every 3 days to maintain cell density < 2 × 10^6^ cells/mL.

### MTT reduction assay

The reference L-929 mouse fibroblasts and guinea pig primary gastric epithelial cells obtained from the specimen of guinea pig gastric tissue were grown as described above. Before being used in the experiments the cell viability and cell density were assessed by trypan blue exclusion assay using a counting Bürker chamber (Blaubrand, Wertheim, Germany). The cells were used in the experiments only if cell viability was higher than 95%. Biocompatibility of BCG alone was determined according to the ISO norm 10993-5 (International Organization for Standardization, 2009; Biological evaluation of medical devices-Part 5: Tests for in vitro cytotoxicity), based on the 3-(4,5-dimethylthiazol-2-yl)-2,5-diphenyltetrazolium bromide-MTT (Sigma-Aldrich, St. Louis, MI, USA) reduction assay, as previously described^56^. The L-929 fibroblasts or primary gastric epithelial cells were seeded (2 × 10^5^ cells/ well) in 96-well culture plates (Nunclon Delta Surface, Nunc, Rochester, NY, USA), and incubated overnight prior to use. Cell morphology and confluency were controlled using an inverted contrast phase microscope (Motic AE2000, Xiamen, People's Republic of China). Suspension of BCG mycobacteria was distributed to the wells of culture plates (MOI 10:1), containing cell monolayers (in 3 replicates). After 24 h incubation, the condition of cell monolayers was verified under inverted contrast phase microscope. The cell cultures in medium without the mycobacteria were used as a positive control of cell metabolic activity whereas the cell cultures in 0.03% H_2_O_2_ served as negative control. To quantify the metabolic activity of cells, 20 µL of MTT was added to each well and incubation was carried out for the next 4 h. The plates were centrifuged at 450 × g for 10 min, the supernatant was removed and the formazan crystals were dissolved with 100 µL dimethyl sulfoxide (Sigma Aldrich, St. Louis, MI, USA). The absorbance was determined spectrophotometrically using the Multiskan EX reader (Thermo Scientific, Waltham, MA, USA), at 570 nm. The effectiveness of MTT reduction was calculated based on the following formula: MTT reduction relative to untreated cells (%) = (absorbance of treated cells/absorbance of untreated cells × 100%)−100%.

### MUC5AC detection in guinea pig primary gastric epithelial cells or gastric tissue

Detection of MUC5AC in guinea pig primary gastric epithelial cells or gastric tissue was performed as previously described^12^. The gastric epithelial cells (1 × 10^6^ cell/mL, 1 mL) were cultured in wells of a 6-well culture plates containing glass coverslips (Thermo Scientific, Waltham, MA, USA), and exposed for 24 h to bacterial antigens: *H. pylori* GE 10 μg/mL, *H. pylori* LPS 25 ng/mL, or control *E. coli* LPS 25 ng/mL, in the milieu without onco-BCG mycobacteria or with mycobacteria in MOI 10: 1 (2 h). We also used cells, which were first primed for 2 h with onco-BCG mycobacteria MOI 10:1, and then with the above-mentioned bacterial antigens for 24 h. Cells were fixed with 4% formaldehyde solution, 20 min, room temp., and then washed 3 times in phosphate buffered saline (PBS). For increasing cell permeability cells were treated with 0.02% Triton-X-100 for 10 min and then washed 3 times as before. After blocking a cell matrix (cover slips) with 3% BSA in PBS, 1 h, cells were used for further procedures. Gastric tissue-*antrum* part of the stomach was collected from control animals or animals 7 and 28 days after the last dose of *H. pylori* alone, onco-BCG mycobacteria alone or BCG vaccine mycobacteria and then *H. pylori*. Gastric tissue-*antrum* part of the stomach specimens was prepared for staining as previously described^12^. In brief specimens were fixed in formalin solution, embedded in paraffin and tissue sections were prepared (4 μm), then placed on the adhesive slide and deparaffinized. For antigen exposure the sections were heated (95–100 °C) in sodium citrate buffer pH 6.0, 20 min, cooled to room temperature and washed 3 times in PBS. Mucin 5AC in the cell cultures or in gastric tissue was determined by staining with primary mouse monoclonal IgG anti-MUC5AC antibodies cross-reacting with MUC5AC of different mammals, including rodents, 1:100 (MyBiosource, San Diego, USA, recommended to formalin fixed cells or tissue samples, isotype IgG1, clone number 45M1), overnight at 4 °C. After washing 5 times in Tris-Buffered Saline with Tween 20 (TBST), pH 7.4, sheep anti-mouse IgG antibodies conjugated with fluorescein isothiocyanate (FITC) 1: 64 (Thermo Scientific, Waltham, MA, USA), were added for 20 min at room temp. Cell nuclei were stained with 4′,6-diamidino-2-fenilindol (DAPI) solution (2.5 μg/mL), at room temperature. Intensity of fluorescence of primary gastric epithelial cells was measured by using a multifunctional SpectraMax i3 (Molecular Devices, San Jose, CA, USA) reader (excitation 495 nm emission 519 nm). Gastric tissue sections were imaged under confocal microscopy (Leica TCS SPE) at the wavelength for each fluorochrome: FITC (excitation 495 nm emission 519 nm), DAPI (excitation 345 nm, emission 455 nm), at a magnification for gastric tissue: 10× or 40× .

### Adhesion of *H. pylori* or onco-BCG to guinea pig primary gastric epithelial cells or gastric tissue

Before using in adhesion assay onco-BCG mycobacteria were fluorescently labelled with LIVE/DEAD BacLight (Thermo Scientific, Waltham, MA, USA). Per 1 mL of the mycobacterial suspension, 3 µL of the reagent were added, and incubation was carried out in the dark for 15 min. The suspension of live *H. pylori* rods was prepared as previously described^12^.

For adhesion assay, the guinea pig primary gastric epithelial cells were treated with bacterial suspensions (MOI 10:1) in the following variants: *H. pylori* alone or BCG alone (2 h), firstly *H. pylori* (2 h) and next BCG (2 h), firstly BCG (2 h) and next *H. pylori* (2 h), simultaneously *H. pylori* and BCG (2 h), in the conditions of incubator (37 °C, 5% CO_2_). Then unbound bacteria were washed twice in PBS. Cells were fixed and stained with anti-*H. pylori* antibodies, DAPI (cell nuclei) (Thermo Fisher Waltham, USA), and TexasRed phalloidin (actin) (Thermo Fisher, Waltham, USA), as previously described^12^. Bacterial adhesion was quantified by measuring the intensity of fluorescence by using a multifunctional SpectraMax i3 (Molecular Devices, San Jose, CA, USA) reader (excitation 495 nm emission 519 nm). Cells were imaged under confocal microscopy (Leica TCS SPE), at the wavelength for each fluorochrome: FITC (excitation 495 nm emission 519 nm), DAPI (excitation 345 nm, emission 455 nm) and phalloidin (excitation 591 nm emission 608 nm), at a magnification of 64×.Three independent experiments were performed in triplicate for each variant.

The thin layer sections of gastric tissue from a guinea pig, which did not receive bacterial suspension, were prepared for adhesion as previously described^12^. Fluorescently stained onco-BCG mycobacteria and/or live *H. pylori* were applied on tissue sections (1 mL of prepared bacterial suspension containing live *H. pylori*-2 × 10^7^ CFU/mL or onco-BCG mycobacteria-1 × 10^8^ CFU/mL). The following variants of tissue sections were used: treated with *H. pylori* or onco-BCG mycobacteria (2 h or 4 h), firstly with *H. pylori* (2 h) and next with onco-BCG mycobacteria (2 h), firstly with onco-BCG mycobacteria (2 h) and next with *H. pylori* (2 h), or simultaneously with *H. pylori* and onco-BCG mycobacteria (2 h and 4 h). All tissue sections were incubated in conditions of incubator (37 °C, 5% CO_2_). Unbound bacteria were washed twice in PBS. Tissue sections were fixed and stained with fluorescently labeled anti-*H. pylori* antibodies and DAPI (cell nuclei) as previously described^12^. First cells were incubated for 1 h with rabbit anti-*H. pylori* antibodies-FITC (100 μL, 1:200 in BSA/PBS (MyBiosource, San Diego, USA). Then tissue were stained with DAPI solution (2.5 μg/mL) 15 min at room temp. Slides were mounted in a DPX balsam (Surgipath, Great Britain) and then imaged in a confocal microscope (Leica TCS SPE) at a wavelength for FITC 495 nm (excitation), 519 nm (emission) and for DAPI 358 nm (excitation), 461 nm (emission), at magnification 10× and 100×. In all experiments, controls of antibodies were set to exclude nonspecific reactions.

### Statistical analysis

Data were expressed as the mean ± standard error of mean (SEM). The differences between groups were tested using the non-parametric Mann–Whitney *U* test or the Kruskal–Wallis test. For statistical analysis the Statistica 13.3 PL (https://statistica.software.informer.com/13.3software) (Krakow, Poland) software was used. Results were considered statistically significant when *p* < 0.05.

## Data Availability

All data generated or analyzed during this study are included in this published article.
